# Coronary computed tomography angiography in primary care patients with chest pain or dyspnea – a cross-sectional study

**DOI:** 10.1186/s12875-025-02877-z

**Published:** 2025-05-20

**Authors:** Erik Stertman, Fade Gabro, Mårten Sandstedt, Oleg Sysoev, Jörg Lauermann, Carl Johan Östgren, Sofia Sederholm Lawesson, Jan Engvall, Staffan Nilsson, Fredrik Iredahl

**Affiliations:** 1 Primary Health Care Center, Department of Health, Medicine and Caring Sciences, Faculty of Medicine and Health Sciences, Linköping University, Universitetssjukhuset, Linköping, 581 83 Sweden; 2 Department of Radiology in Linköping, Department of Health, Medicine and Caring Sciences, Linköping University, Linköping, Sweden; 3 Center for Medical Image Science and Visualization (CMIV), Linköping University, Linköping, Sweden; 4 Department of Computer and Information Science, Linköping University, Linköping, Sweden; 5 Department of Cardiology, Jönköping, Region Jönköping County, and Department of Health, Medicine and Caring Sciences, Linköping University, Linköping, Sweden; 6 Department of Cardiology, Department of Health, Medicine and Caring Sciences, Linköping University, Linköping, Sweden; 7 Department of Clinical Physiology in Linköping, Department of Health, Medicine and Caring Sciences, Linköping University, Linköping, Sweden; 8 Wallenberg Centre for Molecular Medicine, Linköping University, Linköping, Sweden

## Abstract

**Aims:**

Coronary Computed Tomography Angiography (CCTA) is recommended as a first-line investigation to exclude significant coronary artery stenosis in case of low to intermediate pre-test probability (PTP). The aim was to investigate CCTA findings in relation to the PTP of patients referred directly from primary health care centres.

**Methods/Results:**

In this retrospective cohort study consecutive primary care CCTA referrals in a Swedish county 1st of June 2021 until 30th Nov. 2022 were included. CCTA reports were obtained for 483 patients ≥ 30 years old, without known CAD and stratified as no CAD, with atheromatosis or with suspected significant stenosis. For the 381 patients with eligible PTP data, the mean age was 60 years and 70% were women. While the median PTP was 11%, significant stenosis was suspected on CCTA in 18%. Among patients with PTP ≤ 15%, CCTA exposed no significant stenosis in 88%. No significant stenosis was found in patients with PTP < 5% true to patient age and gender in a sensitivity analysis (*n* = 25).

**Conclusions:**

CCTA ruled out coronary stenosis as the cause of chest pain and dyspnea in 88% of patients referred from primary care with PTP 5–15%. PTP estimations by primary care physicians in CCTA referrals agreed with the occurrence of suspected significant stenosis among patients with PTP 5–15%, but underestimated it in PTP > 15%. The validity of PTP estimates < 5% was low.

**Supplementary Information:**

The online version contains supplementary material available at 10.1186/s12875-025-02877-z.

## Introduction

Chest pain and dyspnea are common reasons for consultation in primary health care centres (PHCCs) [1, 2] and may be manifestations of obstructive coronary artery disease (CAD) [3, 4]. Observational studies suggest that symptoms of CAD are insufficiently investigated from primary care, with 20–30% higher risk of major adverse events among patients with no or inconclusive cardiac testing [5, 6], and those diagnosed with chest pain not specified during 6 months [7]. The conventional exercise test is generally the most accessible investigation from primary care. However, the exercise test merely achieves moderate diagnostic sensitivity and specificity [8] and has in studies returned about one in five as inconclusive [6, 9]. A minority of patients referred to cardiology centres for chest pain have a cardiac origin identified [10, 11].

Coronary Computed Tomography Angiography (CCTA) is recommended as a first-line investigation of non-acute chest pain and dyspnea with low-intermediate pre-test probability (PTP) of underlying obstructive CAD [3]. CCTA enable rule out significant coronary stenosis with excellent precision in this population [12]. CCTA referred to from cardiology centres has been thoroughly evaluated, with suspected significant stenosis found in 14–25% of patients with non-acute symptoms [13–15]. In a large Scottish trial obstructive CAD was suspected in 25% among patients randomised to undergo CCTA in patients referred to cardiology centres from PHCCs for chest pain assessment [16]. To our knowledge, the performance of direct referral to CCTA from PHCCs has only been explored in a cross-sectional study of patients recruited at a hospital-associated PHCC in Ontario. In that study 14% of the 148 study participants had suspected significant stenosis [17]. The diagnostic yield from CCTA is likely to vary depending on whether patients are selected from a primary or specialized care setting [18–20].

This study aimed to evaluate the CCTA findings among patients directly referred from PHCCs and in relation to pre-test probability (PTP) of obstructive CAD and cardiovascular (CV) risk factors. We hypothesized that among patients referred with PTP ≤ 15% for obstructive CAD, this could be ruled out in at least 85% of patients indicating alignment with the rates employed in the ESC 2019 guidelines. We also hypothesized that the prevalence of suspected significant stenosis would be on par with the median PTP.

## Methods

This cross-sectional retrospective cohort study included all consecutive clinical referrals for a CCTA from all 44 (March 2022) PHCCs in the Swedish county of Östergötland 1st of June 2021 to 30th of Nov 2022. These PHCCs served approximately 471 000 inhabitants, and 33 centres were under public management while 11 were private. As of 1st of June 2021 the local health authority promoted referral to CCTA from PHCCs as a first-line investigation for chest pain patients with PTP of 5–15%. For patients with PTP < 5%, other causes than CAD should be sought for, but CCTA could be referred to if the primary care physician deemed that CAD must be excluded. For patients with PTP > 15% the exercise test was recommended as a first-line investigation from PHCCs, or a direct referral to a cardiologist in case of strong suspicion and high PTP or if an exercise test was not suitable to perform (pathological rest ECG, knee or hip pain, unable to ride a bike etc.). The local health authority held guideline seminars, but no training sessions, and a written instruction for PTP estimation in-line with the 2019 European Society of Cardiology guidelines [3] was made available. Referrals for more advanced imaging techniques such as single-photon emission computed tomography (SPECT), stress echocardiography or perfusion magnetic resonance imaging (MRI) had to come from a cardiologist. Direct primary care referral for myocardial perfusion imaging was discouraged. The study explored the referral process for CCTA from PHCCs through the referral letters, the radiological reports, and care register data of CV risk factors.

### Study population

We included patients by identifying CCTA referrals from 1st of June 2021 to 30th of Nov 2022 from PHCCs to any of the Radiology Departments at the three hospitals in the County of Östergötland using the local radiology information system (RIS).

**Fig. 1:**
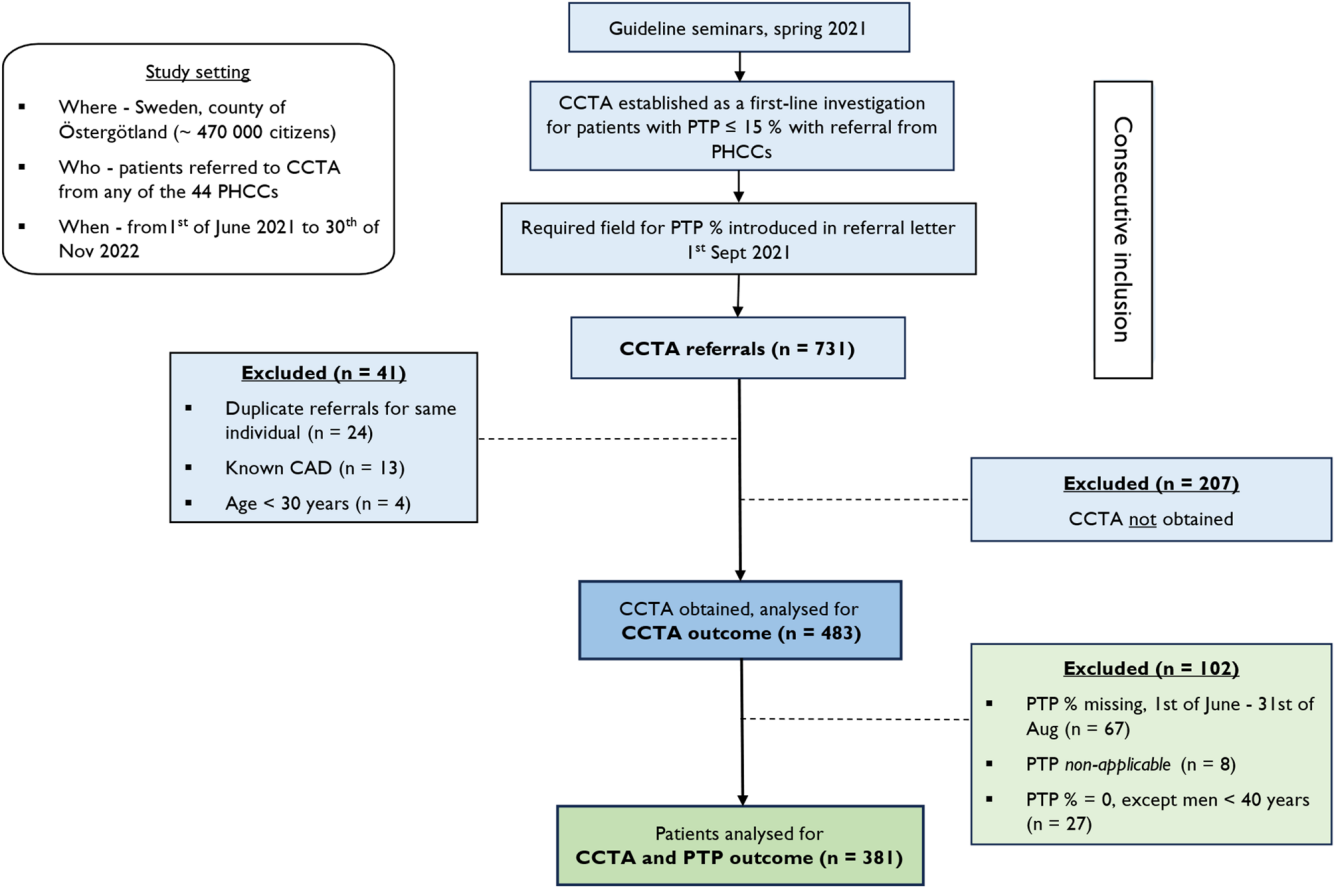
Flowchart of inclusion, exclusion, and patients eligible for analysis. The study included patients by date of referral to coronary computed tomography angiography (CCTA) from primary health care centres (PHCCs). At study closing 207 patients had not obtained a CCTA in the county, a majority of whom had been referred within 90 days and 81% with pre-test probability (PTP) > 5%

As outlined in Fig. 1, duplicate referrals for the same personal identity number, previous CAD (percutaneous coronary intervention [PCI], coronary artery by-pass grafting [CABG] or myocardial infarction [MI]), and patients aged < 30 years (not included in the ESC 2019 PTP table) were excluded. The study population was established by exclusion of the following referrals to CCTA, in order: CCTA not obtained during the inclusion period, missing PTP, PTP set to ‘non-applicable*’* and PTP set to ‘0’ except for men < 40 years. PTP ‘0’ is only available in the PTP tables for men 30–39 years of age with dyspnea and occurred unproportionally in relation to that, why we chose to exclude PTP ‘0’ not available for gender or age interval to enable further analysis.

Post-hoc sensitivity analysis was prompted by a higher-than-expected prevalence of suspected significant stenosis among patients referred with PTP < 5%, to investigate the validity of the referred PTP and effects on our hypotheses.

### Image acquisition

The CCTAs were performed at three hospitals using five types of Siemens CT scanners, all with ≥ 64 detector rows: Siemens NAEOTOM Alpha, Siemens SOMATOM Force, Siemens SOMATOM Drive, Siemens SOMATOM Definition Flash, and Siemens SOMATOM Edge. A prospectively ECG-gated high-pitch protocol, a sequential mode protocol, or a retrospectively gated spiral protocol was used, depending on patient characteristics and heart rate. Nitrolingual spray (2 × 0.4 mg) and Metoprolol (5–10 mg IV) were administered immediately before scanning, if no contraindications.

### Variables

All CCTA scans had been clinically interpreted by a total of 12 radiologists in three hospitals, producing written reports. From these reports a thoracic radiologist (MS), with 13 years’ experience of CCTA reading, performed a categorization of stenosis severity into: (1) significant stenosis, defined as a suspected ≥ 50% obstruction of any coronary vessel lumen, (2) atheromatosis, defined as presence of coronary vessel wall plaques > 1 mm^2^ with < 50% stenosis, (3) no signs of CAD, defined as absence of atheromatous plaque and no luminal stenosis [21]. The thoracic radiologist (MS) also graded image quality based on information in the clinical reports into three categories: satisfactory, partially inconclusive, or completely inconclusive. Only reports categorized into satisfactory or partially inconclusive were graded for stenosis severity. The stated reasons for inconclusive and partially inconclusive investigations were collected. To validate the clinical reports a second read of 53 randomly selected CCTA scans (11%) were performed by the thoracic radiologist. Of those, there were no disagreements in 45 CCTA scans. Possible disagreements (*n* = 8, 15%) were presented to an independent thoracic radiologist, who regarded seven as ambiguous or with no clinical impact, and one shifted from ‘completely inconclusive’ to ‘partially inconclusive’ with ‘atheromatosis’, of which the referring physician was informed.

A PTP % from the referring physician was collected through the CCTA referral letter. The referrals from 1st June to 31st Aug 2021 were excluded from the study if PTP % was missing in the medical history as described in the referral. For the remainder of the study period, the electronic referral template included a mandatory drop-down menu with options of the percentage figures of PTP available in Supplementary Fig. 1, starting with ‘0’ at the top and at the bottom ‘52’ and ‘not applicable’ (in Swedish).

PTP is recommended to be estimated based on the main symptom, either chest pain or dyspnea. Availability of the referred PTP in the dyspnea PTP table (see Supplementary Fig. 1) was used to code the main symptom. If the given PTP % was unavailable in the dyspnea table, the main symptom was set to chest pain. As chest pain and dyspnea tables overlap for women 30–39 years (PTP 3%), these were assessed based on additional information in the referral text. Age (at the date of the referral) and legal gender were derived from the Swedish personal identity number (possible to change according to gender identity, hence “gender” and not biological “sex” for clarity). The referral texts were reviewed for indications that the primary care physician consulted a cardiologist preceding CCTA referral and entry of a PTP estimation.

### Data sources

Patient data from the referrals and the radiology reports were retrieved from the local RIS and Picture Archiving and Communication System (PACS) of the Radiology Clinics of Region Östergötland. SWEDEHEART, a nationwide quality registry for coronary artery disease (CAD), was used after the inclusion period to retrieve data regarding: previous percutaneous coronary intervention (PCI), coronary angiography by-pass grafting (CABG), previous myocardial infarction (MI), diabetes mellitus (type 1 and type 2), smoking, hypertension (at least one anti-hypertensive medication), lipid-lowering medications, body mass index (BMI, derived from registered body height and body weight), estimated glomerular filtration rate (eGFR, creatinine clearance by Cockcroft-Gault) [22]. These SWEDEHEART data were registered for patients at the time of the CCTA examination. For this study, we used a local part of the SWEDEHEART registry where CCTA examinations were collected. A database was created by linking CCTA referrals to radiology reports and the SWEDEHEART data.

### Statistical analysis

Descriptive statistics is presented as numbers and percentages, and continuous variables as mean ± standard deviation (SD) or median with interquartile range (IQR) as appropriate. As PTP figures represent the discrete values present in the ESC 2019 guidelines PTP tables, and are of ordinal type, medians with interquartile ranges (IQR) will be presented. For the subgroup analysis, the PTP % was categorized into < 5, 5–15 and > 15 advocated for in the ESC 2019 guidelines. Comparisons between PTP groups and CCTA outcome categories were made using Chi-square test for categorical data, one-way ANOVA for continuous data which met assumption of normal distribution (i.e. creatinine clearance) and Kruskal-Wallis test for two groups and Mann-Whitney test for more than two groups of PTP %. Pairwise comparisons among groups of three were performed using Chi-square test or paired T-test. Family-wise type I error was avoided using the p-value from the comparison of all three groups if greater than the pairwise value [23]. The mean effective radiation dose was calculated from the mean dose length product multiplied by k = 0.014. Values of zero for variables such as BMI and eGFR were considered missing and removed from further analysis. These data are presented in Supplementary Table 1. Post-hoc sensitivity analysis was performed by creating a subset of referrals with eligible PTP (*n* = 381) by only including those with figures of PTP consistent with patient legal gender and age group in Supplementary Fig. 1 (i.e. with either dyspnea or chest pain and any estimation of chest pain characteristics). Difference in distribution for PTP was tested with Mann-Whitney test. In all analyses a p-value < 0.05 was considered statistically significant. Data analysis and statistical computations were performed using IBM-SPSS Statistics v. 29.0.0.0 (IBM Corporation, Armonk, NY, USA).

## Results

### Participants

The study included 731 primary care referrals for CCTA. Reports were available for 483 patients, mean age of 60 years (SD 12), 68% women (group characteristics and CCTA findings are presented in Supplemental Table 2). Among the 381 patients with eligible PTP figures (Fig. 1), who constituted the main study population, the mean effective radiation dose was 5.3 (SD 4.1) mSv, mean age was 60 years (SD 11), 70% were women, mean BMI was 28 (SD 5), 12% had diabetes, 47% had hypertension, 30% had lipid-lowering medication, 13% were active smokers and the mean eGFR was 93 mL/min per 1.73 m^2^ (SD 33). A physician-to-physician consultation with a cardiologist was noted in 24% of the cohort.

### CCTA findings and overall performance

In the main study cohort of 381 patients, suspected significant stenosis was detected in 18.1% (*n* = 69), atheromatosis in 31.0% (*n* = 118), no signs of CAD in 50.4% (*n* = 192), while 0.5% (*n* = 2) CCTA scans were inconclusive. In addition to the two completely inconclusive CCTA scans, 39 were partially inconclusive. These constituted 15% (*n* = 10) of patients with suspected significant stenosis, 14% (*n* = 17) with atheromatosis and 6% (*n* = 12) with no signs of CAD (*p* = 0.03). High or irregular pulse was the most frequently reported cause of partially or completely inconclusive scans in all CCTA categories, ranging from 29 to 40%. In Supplementary Table 2, the patients factor associations to CCTA findings are presented.

### CCTA findings in relation to median PTP and main symptom

Among the 381 patients the median PTP was 11% (IQR 9). Among patients referred with a PTP indicating dyspnea as the main symptom (*n* = 72), the prevalence of suspected significant stenosis was 9.7%, whereas it was 20.1% for patients with predominantly chest pain (*n* = 309). The median PTP was 11% (IQR 10) for patients with chest pain and 12% (IQR 5) for dyspnea patients (*p* > 0.05). For patients with suspected significant stenosis, the median PTP was 16% (IQR 13.5) for patients with chest pain and 14% (IQR 2) for patients with dyspnea (*p* > 0.05). The patient characteristics per subset of main symptom is presented separately in Table 1.

**Table 1:** Subsets per main symptom as derived from the PTP estimated by referring primary care physician

	Chest pain (*n* = 309)	Dyspnea (*n* = 72)	Difference in distribution, *p*-value^e^
Suspected significant stenosis, No (%)	62 (20)	7 (10)	0.40
Atheromatosis, No (%)	89 (29)	29 (41)	0.58
No CAD, No (%)	157 (51)	35 (49)	0.74
PTP %, median (IQR)	11 (10)	12 (5)	Not applicable^e^
PTP < 5%, No (%)	50 (16)	2 (3)	Not applicable^e^
PTP 5–15%, No (%)	170 (55)	64 (89)	Not applicable^e^
PTP > 15%, No (%)	89 (29)	6 (8)	Not applicable^e^
Years of age, mean (SD)	59 (11)	64 (11)	< 0.001
Women (vs. men), No. (%)	208 (67)	59 (92)	0.015
BMI, mean (SD)^a^	28 (5)	29 (5)	0.15
Diabetes mellitus, No. (%)^b^	39 (13)	7 (10)	0.50
Hypertension, No. (%)^c^	139 (45)	39 (54)	0.18
Lipid-lowering drug, No. (%)	93 (31)	20 (28)	0.65
Smoking, current, No. (%)	44 (14)	7 (10)	0.31
Smoking, previous, No. (%)^d^	111 (36)	33 (46)	0.12
Creatinine clearance, mean (SD)^a^	73 (16)	75 (15)	0.18

^a^ Excluding values of zero, BMI (kg/m^2^) and creatinine clearance (mL/min per 1.73 m^2^)

^b^ Type I and II.

^c^ At least one blood pressure lowering drug.

^d^ Stopped smoking more than one month ago.

^e^ Tested with Pearsons’s Chi2, except for variables Years of age, BMI, Creatinine clearance which were tested by one-way ANOVA.

^e^ Variables does not meet assumption of independence.

### Characteristics per PTP group

In Table 2 the demography, the prevalence of cardiovascular risk factors and the rate of cardiologist consultation are presented according to PTP group. The referral PTP was 5–15% for 61% of patients who completed CCTA. There was no difference among PTP groups in partially or completely inconclusive examinations (12%, 11% and 10%, *p* = 0.9).

**Table 2:** Cardiovascular risk profile, main symptom and cardiologist consultation rate according to PTP group

	PTP (%) from referring physician *n* = 381			Difference in distribution
PTP groups, No. patients (%)	**< 5** 52 (14)	**5–15** 234 (61)	**> 15** 95 (25)	**p-value** ^g^
Years of age, mean (SD)	55 (13)	59 (11)	64 (10)	0.004
Women, No. (%)	34 (65)	192 (82)	41 (43)	< 0.001
BMI, mean (SD)^a^	27 (5)	29 (5)	28 (5)	0.394
Diabetes mellitus, No. (%)^b^	9 (17)	21 (9)	16 (17)	0.099
Hypertension, No. (%)^c^	21 (40)	99 (42)	58 (61)	0.004
Lipid-lowering drugs, No. (%)	16 (31)	61 (26)	36 (38)	0.082
Smoking, current, No. (%)	9 (18)	29 (12)	13 (14)	0.606
Smoking, previous, No. (%)^e^	19 (37)	87 (37)	38 (40)	0.874
Creatinine clearance, mean (SD)^a^	96 (17)	95 (14)	87 (17)	0.259
Dyspnea PTP %, No. (%)^e^	2 (4)	64 (27)	6 (6)	< 0.001
Cardiologist consultation, No. (%)	17 (33)	31 (14)	41 (44)	< 0.001

^a^ Excluding values of zero, BMI (kg/m^2^)(*n* = 3), creatinine clearance (mL/min per 1.73 m^2^)(*n* = 118)

^b^ Type I and II

^c^ At least one blood pressure lowering drug

^d^ Stopped smoking more than one month ago

^e^ PTP value in the dyspnea table from the ESC 2019 guidelines on chronic coronary syndrome (Knuuti 2019), main symptom considered as chest pain for the remainder of PTP values

^g^ Tested with Pearsons’s Chi2, except for variables Years of age, BMI, Creatinine clearance which were tested by one-way ANOVA

### CCTA findings per PTP group

**Fig. 2:**
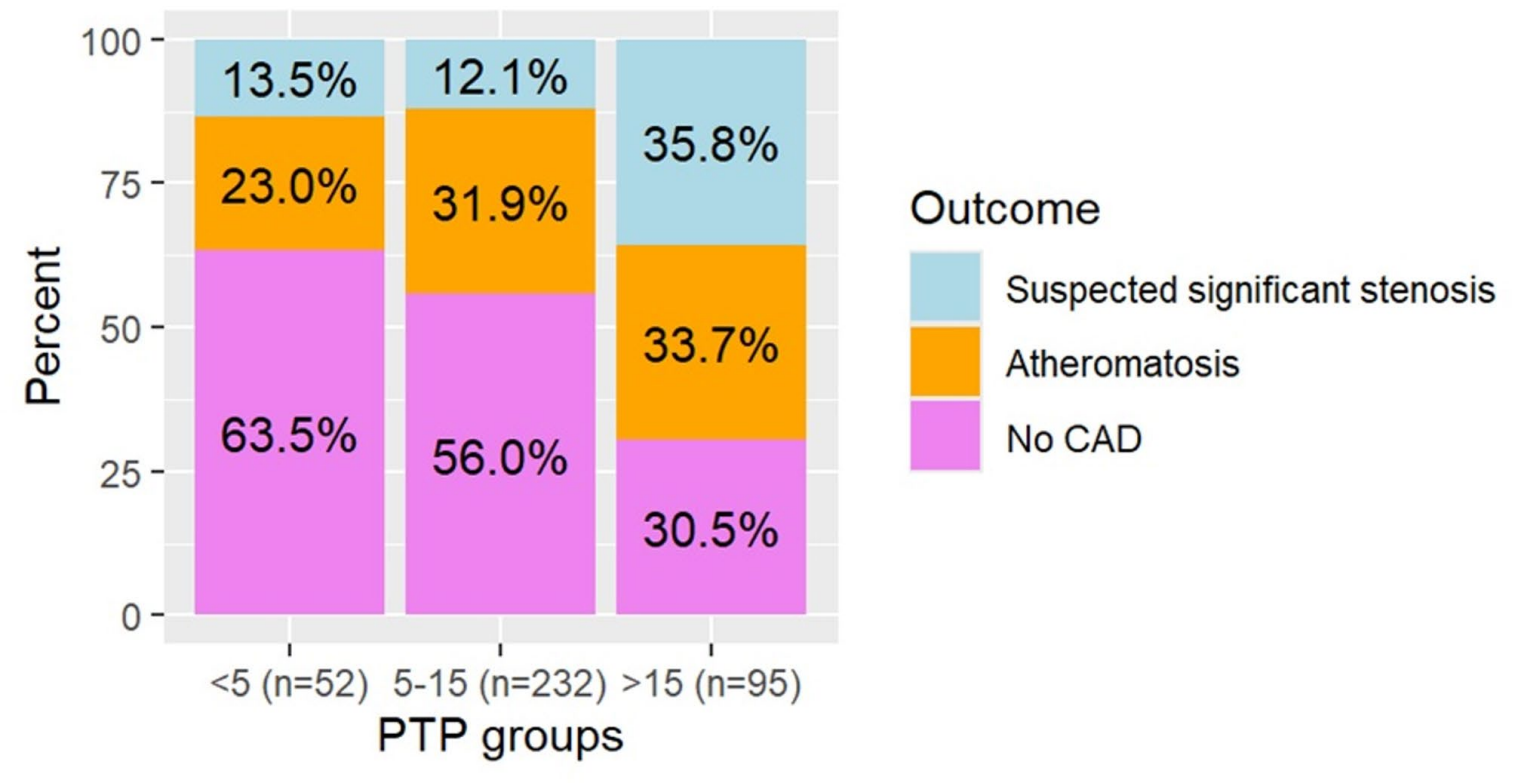
Coronary computed tomography angiography (CCTA) findings per group of pre-test probability (PTP) estimated by the referring primary care physician. Of the 381 patients with eligible PTP and completed CCTA, two had inconclusive scan results and are not shown in the figure. Bars represent ‘no CAD’ (coronary artery disease, bottom), atheromatosis (middle) and suspected significant stenosis (≥ 50% occlusion, top)

The prevalence of suspected significant stenosis in patients with PTP ≤ 15% was 12.3%. Accordingly, 87.7% of the group had either atheromatosis without significant stenosis, no signs of CAD or inconclusive examinations. In Fig. 2 the CCTA result per PTP group (*n* = 379) is presented. Subgroup analysis indicated significant differences between PTP groups in the occurrence of no CAD (*p* < 0.001) and in suspected significant stenosis (*p* < 0.001), while there was no difference in the presence of atheromatosis without significant stenosis (*p* = 0.39). As compared to the group with PTP < 5% suspected significant stenosis was more prevalent in the PTP 5–15% as well as in the PTP > 15% group (*p* = 0.004 and *p* < 0.001, respectively). No difference was found between the two latter groups (*p* > 0.05). The absence of CAD was significantly higher in the PTP < 5% compared to the PTP 5–15% and the PTP > 15% (*p* < 0.001, both comparisons), but not between the latter groups (*p* > 0.05).

The median PTP % differed between the CCTA outcome groups group (*p* < 0.001); 10 (IQR = 7) in the group with no signs of CAD, 11 (IQR = 7) in the atheromatosis group and 14 (IQR = 12) in the suspected significant stenosis. The median PTP % was 3 (IQR = 1) for the PTP < 5% group, 10 (IQR = 3) for the 5–15% group and 20 (IQR = 10) for the group with PTP > 15%.

### Sensitivity analysis subset according to PTP group

The subset of patients with PTP figures consistent with age group and gender constituted 83% (*n* = 315) of referrals included in the main analysis (*n* = 381). The median PTP was 11% (IQR = 7) and the overall distribution similar to the main analysis (*p* = 0.25). In the subset, 18% had a suspected significant stenosis, among which the median PTP was 16% (IQR 16).

**Fig. 3:**
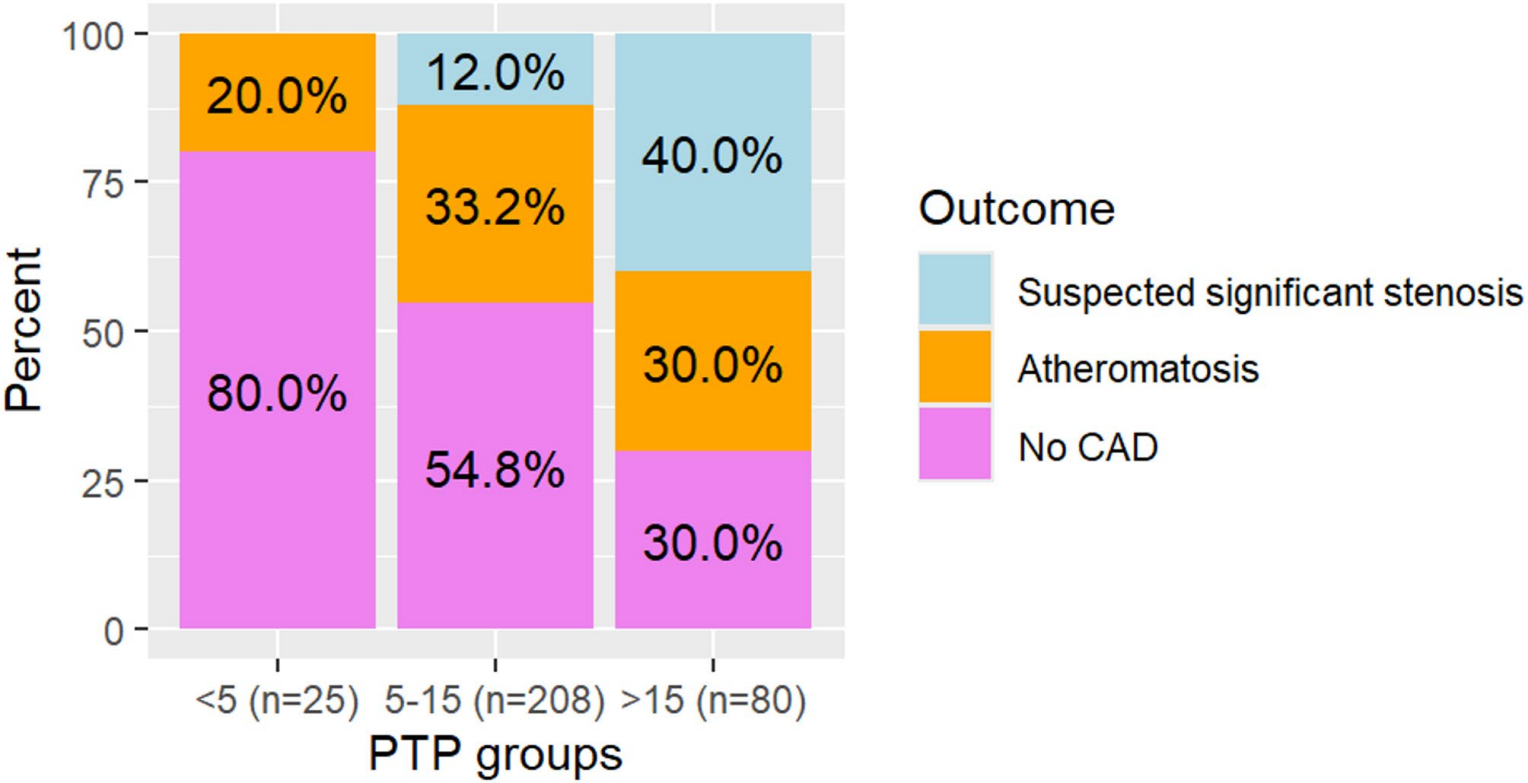
Subset analysis (*n* = 315, two inconclusive CCTA with PTP 5–15% not shown) of CCTA findings per group of PTP for patients referred with figures of PTP consistent with gender and age interval in the PTP tables published by the European Society of Cardiology in 2019. PTP figures *not* consistent with either were excluded post-hoc (*n* = 66) from the main analysis group (*n* = 381). Bars represent ‘no CAD’ (coronary artery disease, bottom), atheromatosis (middle) and suspected significant stenosis (≥ 50% occlusion, top)

Subset CCTA results and patient characteristics are presented in Fig. 3; Table 3, according to PTP group. Patients remaining in the subset compared to the main analysis group according to PTP group was 48%, 90% and 84%. In the PTP groups the median PTP was 3% (IQR = 1), 11% (IQR = 4) and 21% (IQR = 10), which did not differ to the corresponding PTP groups in the main analysis (*p* > 0.05). Among patients with PTP ≤ 15% (*n* = 233), significant stenosis was suspected in 10.7% and for two patients the CCTA was deemed as inconclusive. For PTP 5–15%, a suspected significant stenosis was found in 1 of 43 patients aged 30–49 years, and in 8 of 81 aged 50–59 years.

**Table 3:** Sensitivity analysis subset according to PTP group

	PTP% from referring physician (*n* = 315)			Difference in distribution
PTP groups, No. patients (%)	**< 5** 25 (8)	**5–15** 210 (67)	**> 15** 80 (25)	**p-value** ^f^
Years of age, mean (SD)	46 (7)	58 (11)	65 (9)	< 0.001
Women (vs. men), No. (%)	18 (72)	173 (82)	33 (41)	< 0.001
BMI, mean (SD)^a^	27 (6)	28 (5)	28 (5)	0.66
Diabetes mellitus, No. (%)^b^	2 (8)	19 (9)	10 (13)	0.64
Hypertension, No. (%)^c^	7 (28)	87 (42)	50 (63)	< 0.001
Lipid-lowering drug, No. (%)	4 (17)	53 (26)	31 (39)	0.03
Smoking, current, No. (%)	6 (25)	26 (12)	12 (15)	0.231
Smoking, previous, No. (%)^d^	8 (32)	79 (38)	30 (38)	0.857
Creatinine clearance, mean (SD)^a^	111 (40)	95 (34)	86 (28)	0.001
Dyspnea PTP %, No. (%)^e^	2 (8)	52 (25)	4 (5)	< 0.001
Cardiologist consultation, No. (%)	8 (33)	28 (14)	36 (46)	< 0.001

^a^ Excluding values of zero, BMI (kg/m^2^)(*n* = 3), creatinine clearance (mL/min per 1.73 m^2^)(*n* = 98)

^b^ Type I and II

^c^ At least one blood pressure lowering drug

^d^ Stopped smoking more than one month ago

^e^ PTP value in the dyspnea table from the 2019 European Guidelines for the diagnosis and management of chronic coronary syndrome [3], main symptom considered as chest pain for the remainder of PTP values

^f^ Tested with Pearsons’s Chi2, except for variables Years of age, BMI, Creatinine clearance which were tested by one-way ANOVA

## Discussion

The main finding was that 88% of patients with PTP ≤ 15%, investigated by primary care physicians with CCTA as routine management, had no suspected significant stenosis, allowing for the exclusion of obstructive CAD. In the sensitivity analysis subset of patients with PTP < 5% consistent with patient age and gender, no suspected significant stenosis was found. In both the main analysis and the sensitivity analysis, the median PTP of patients referred with PTP 5–15% (10 and 11%, respectively) were similar to the occurrence of suspected significant stenosis (12% and 11%, respectively). Thus, characteristics of this Swedish primary care cohort are in line with the reference cohorts for PTP [3].

Among 381 patients referred from PHCCs, the occurrence of suspected significant stenosis on CCTA (18%) was higher than the median estimated PTP (11%) and these rates were supported by the sensitivity analysis. This gap indicates that patients had higher clinical risk than conferred by the ESC 2019 PTP values as estimated by the primary care physicians. Contributing factors may include the study’s small size, the recommended requirement of at least one CV risk factor in the county guidelines, differences in population selection [24], differences in the use of invasive angiography for stenosis grading [3] and higher CV risk in Sweden [25]. These circumstances limit direct comparison regarding the selection of patients to CCTA between the PHCCs and the reference cohorts for PTP. The underestimation of significant stenosis by PTP is consistent with a Danish register study of CCTAs for non-acute chest pain or dyspnea of patients referred from specialized care [26]. The underestimation was most extensive in patients with PTP > 15% in the sensitivity subset (median PTP 21, suspected significant stenosis 40%). However, this finding may not be generalisable, as CCTA was not the recommended first-line investigation for PTP > 15%, and thus a higher risk population might have been selected, based on limitations of an exercise test or insufficient diagnostic information of a preceding exercise test, or recommendations from a cardiologist.

In our study, 12% of patients referred with PTP 5–15% had suspected significant stenosis (Figs. 2 and 3), which is higher than findings in a Finnish hospital study (4.7%) [27] but lower compared to findings in a Danish region (17.9%) [26]. The variability in findings could be due to differences in patient selection (the recommendation of at least one risk factor, employment of different guidelines, supposed inclusion of suspected acute coronary syndrome in the Finnish study) and the method for estimating PTP (not clearly presented in either study).

Sixty-one percent of our study cohort had PTP 5–15%, in line with local CCTA referral guidelines. Cardiological consultation prior to referral was less common for these patients compared to those with PTP < 5% or > 15% (14% compared to 33% and 44%). This could indicate a shared decision making and the consultation was presumably used as ‘go-ahead’ for referral to CCTA. Referrals with PTP < 5% constituted 14% of the main cohort and 8% of the sensitivity subset. The rate of CCTA examinations for patients with PTP < 5% compared to those with PTP 5–15% was lower in our cohort (22% in the main analysis, 25% when adding the excluded 27 patients with PTP ’0’) compared to the Danish and Finnish cohorts (43% and 49%, respectively) [26, 27]. This suggests that the primary care physicians deferred testing for patients with very low risk or investigated patients with 5–15% more liberally, adhering to guidelines.

In this study the occurrence of suspected significant stenosis was higher among patients with chest pain as main symptom (derived from the estimated PTP), compared to dyspnea (20 vs. 10%). In a previous study from our health care setting on consecutive CCTA referrals for stable chest pain patients from cardiologists, suspected significant stenosis was found in 15% and no CAD in 55%, with a similar mean age (60 vs. 59 years) but a higher proportion of men (43 vs. 33%) [28]. Interestingly, a similar rate of suspected significant stenosis (12% vs. 14%) was found among participants in SCAPIS (the population-based Swedish CArdioPulmonary bioImage Study, randomly invited 50-64-year-olds) who reported angina-like symptoms in a designated questionnaire [29, 30], compared to the corresponding age interval and with chest pain (*n* = 148, Supplementary Table 3).

Chest pain persists over 6 months in more than half of patients after an initial primary care consultation [31] and in those with chronic coronary syndrome [32]. First-line exercise test, with a higher proportion of false negative and inconclusive results compared to CCTA [8], is followed by a deceptive clinical situation in which the risk of major adverse outcome may not be recognized [33]. If CCTA is implemented in primary care, presumably four out of five patients with chest pain or dyspnea will have no significant coronary stenosis which has been associated with a low risk of a major cardiac event (0.32% annually over 2-year median follow-up) [12]. Thus, CCTA may contribute to person-centredness and continuity of care [24, 34], empower gatekeeping [35], and reduce the need for other tests [36]. While in this study a quarter of CCTAs were preceded by cardiological consultation [11], we expect this to decrease with increasing experience.

On the other hand, CCTA first-line is a resource-demanding transition (e.g., time to CCTA increased during the study period, Supplementary Table 4) and entails radiation exposure, while clinically meaningful impact on patient outcomes has not yet been convincingly demonstrated [37, 38]. Intriguingly, the addition of CCTA to standard care among patients referred from primary care to cardiology centres in the SCOT-HEART trial was associated with a 41% lower mortality from CAD or non-fatal myocardial infarction after 5 years, ascribed to higher coverage of lipid-lowering and anti-thrombotic drugs in the group who were randomized to CCTA [9]. Hypothetically, investigation with CCTA from the PHCCs could facilitate an individualized preventive therapy for 17% of the study patients, as lipid-lowering treatment was lacking in 9% with suspected significant stenosis and was prescribed to 8% with no signs of CAD (Supplementary Table 2). In addition, CCTA may allow better risk stratification among the 30% of patients with atheromatosis and no significant stenosis [39]. As medical testing increases healthcare expenditure and drug prescription, these may be important targets for future studies after implementation of direct CCTA referral from primary care.

This study shows the diagnostic yield of direct primary care referral to CCTA as routine management in a Swedish geographical catchment area. The yield broadly matched the estimated PTP from ESC 2019 guidelines and was non-inferior to the compared specialized care cohorts. Since 2024, ESC guidelines advocate for a risk-factor weighted clinical likelihood (RW-CL) to replace the Diamond-Forrester PTP [40]. Although the new and more comprehensive model for estimating the pre-test likelihood remains to be validated among patients in primary care, we found that CCTA with direct referral from PHCCs is feasible for low to intermediate risk of obstructive CAD. The presence of at least one CV risk factor was recommended in the local guideline in addition to PTP 5–15%, reflecting ESC 2019 guidelines on advocacy for CCTA. The rates of CV risk factors, in our study were similar to in the Danish CCTA cohort [26] and to the patients with chest pain referred for assessment of CAD in a Scottish trail [16]. This suggests that the patients referred to CCTA from the PHCCs were clinically selected for higher risk. Furthermore, the comparability of our study findings to those of the Danish CCTA registry, underlying the RW-CL in recent guidelines [41], suggests that it could be efficiently employed in primary care, where patients generally have undergone less selection and present with less specific symptoms [24].

This study has some limitations. PTP was not available for 21% of patients completing CCTA, however this is unlikely to introduce a sampling bias. At least two thirds of referrals without PTP were made during the first three months of the study period when no separate entry for PTP was available in the referral template. While the low validity of clinical PTP estimations < 5% impedes interpretation of the total PTP (as evident from the sensitivity analysis and occurrence of PTP ‘0’ excluded a priori), we observed high validities for PTP estimated to 5–15% and > 15%. There is a paucity of previous data on the validity of clinical PTP estimation, limiting comparisons. Although no specific training in the PTP algorithm was undertaken before the study, the primary care physicians had been offered an introductory seminar on the ESC 2019 guidelines. Speculatively, barriers for accurate PTP estimation in this study includes non-compliance to estimation guidelines, PTP estimations for patients without chest pain or dyspnea, and the design of the digital drop-down menu for entering PTP (showing only low PTP values and PTP ‘0’ at the top, with the possibility to scroll and reveal higher PTPs as well as the ‘non-applicable’ option). For example, in a Danish CCTA register study 20% of patients had no chest pain or dyspnea (thus no PTP), which is comparable to the sum total of referrals with PTP ‘0’, ‘non-applicable’ and impossible PTP figures (24% of 416 referrals) [26]. Accordingly, measures aimed at supporting physicians in using the PTP estimation algorithm in the clinic could have improved the overall validity of PTP. Interventions to increase structure and accessibility of guidelines in the referral process have been associated with higher quality of referrals [42, 43], which in this context could be to allow for separate entries of the factors contributing to PTP.

In addition, the selection of patients for CCTA during the study period reflected the clinical judgement of the physicians involved in patient care, as per the observational study design, which limits conclusions about patient flow. The study design did not involve a control group, why we are unable to make comparisons to other courses of action in the primary care were CCTA not available as a first-line test for direct referral. Since clinical data was used, radiologists were not blinded to patient characteristics nor PTP. While classification of CCTA findings (and quality grading) were based on the reports, the second read validation indicated excellent agreement with the reports from the 12 radiologists with varied reader experience. The inclusion of partially inconclusive CCTA examinations may have impacted the analysis results, however their inclusion contributes to broader conclusions on the real-life applicability of CCTA in primary care. While the proportion of inconclusive CCTAs (0.4%) was low compared to other low to intermediate risk cohorts (4–5%) [9, 27], the rate of both partially and completely inconclusive examinations were comparable to a previous study conducted in the same health care setting (10.8 vs. 10.5%) [28].

## Conclusions

Direct referral to CCTA from primary care resulted in the ruling out of significant coronary stenosis in 88% of patients with PTP 5–15%. The median PTP in this group matched the occurrence of suspected significant stenosis. When PTP was estimated to < 5% and in accordance with patient age and gender, no significant coronary stenosis was found. Direct referral to CCTA from primary care produced an efficient patient selection.

## Electronic supplementary material

Below is the link to the electronic supplementary material.

Supplementary Material 1

Supplementary Material 2

Supplementary Material 3

Supplementary Material 4

Supplementary Material 5

## Data Availability

The data will be shared on reasonable request to the corresponding author.

## Glossary

CABG: Coronary artery by-pass grafting.
CAD: Coronary artery disease.
CCTA: Coronary artery computed tomography angiography.
CV: Cardiovascular.
MI: Myocardial infarction.
MRI: Magnetic resonance imaging.
PCI: Percutaneous coronary intervention.
PHCC: Primary health care centres.
PTP: Pre-test probability.
RIS: Radiology information system.
SCAPIS: Swedish cardiopulmonary bioimage study.
SCOT-HEART: Scottish computed tomography of the heart.
SPECT: Single-photon emission computed tomography.
SWEDEHEART: Swedish web-based system for enhancement and development of evidence-based care in heart disease evaluated according to recommended therapies.
