# Supplementary material for: Coronary computed tomography angiography in primary care patients with chest pain or dyspnea – a cross-sectional study
Source: BMC Prim Care. 2025 May 20;26:178. doi: 10.1186/s12875-025-02877-z (PMC12090552; doi:10.1186/s12875-025-02877-z)

### Supplementary Figure 1. Pre-test probabilities for significant coronary stenosis, adapted from Knuuti et al (2019)(3)


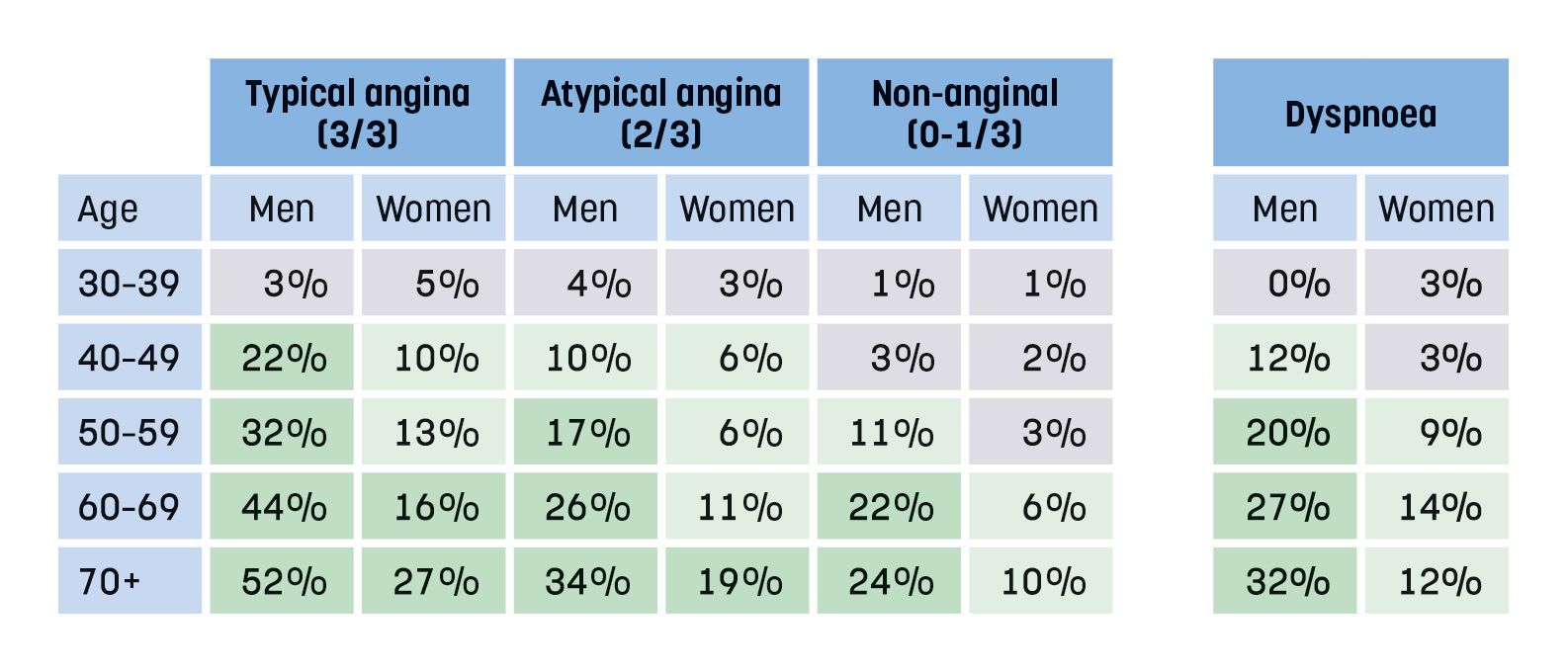

Supplement: Supplementary file 1 — Supplementary Material 1 [file 12875_2025_2877_MOESM1_ESM.docx]
