# Supplementary material for: Coronary computed tomography angiography in primary care patients with chest pain or dyspnea – a cross-sectional study
Source: BMC Prim Care. 2025 May 20;26:178. doi: 10.1186/s12875-025-02877-z (PMC12090552; doi:10.1186/s12875-025-02877-z)
Supplement: Supplementary file 2 — Supplementary Material 2 [file 12875_2025_2877_MOESM2_ESM.docx]

### Supplementary Table 4. Comparison of patients with and without coronary computed tomography angiography (CCTA) report during study period and time from referral to CCTA or study closing

|  | **All CCTA not obtained**  **(n = 207)** | **CCTA not obtained with eligible PTP** | | | **CCTA obtained (n = 483)** |
| --- | --- | --- | --- | --- | --- |
|  |  | **PTP < 5**  (n = 21) | **PTP 5 – 15** (n = 122) | **PTP > 15** (n = 46) |  |
| Years of age, mean (SD) | 60 (11) | 49 (11) | 60 (10) | 67 (10) | 60 (12) |
| Women (vs. men), No. (%) | 147 (71) | 12 (57) | 103 (84) | 21 (46) | 329 (68) |
| Cardiologist consultation, No. (%) | 47 (23) | 7 (33) | 21 (17) | 15 (33) | 124 (26) |
| Days from referral to study closing or CCTA, median (IQR) | 114 (112) | 67 (160) | 63 (125) | 98 (161) | 75 (64) |
