# Supplementary material for: Coronary computed tomography angiography in primary care patients with chest pain or dyspnea – a cross-sectional study
Source: BMC Prim Care. 2025 May 20;26:178. doi: 10.1186/s12875-025-02877-z (PMC12090552; doi:10.1186/s12875-025-02877-z)
Supplement: Supplementary file 3 — Supplementary Material 3 [file 12875_2025_2877_MOESM3_ESM.docx]

### Supplementary Table 2. CCTA results and patient factor associations according to CCTA finding

|  | **CCTA findings category***  **(n = 481)** | | | **Difference in distribution** |
| --- | --- | --- | --- | --- |
|  | **Suspected significant stenosis (19%, n = 90)** | **Atheromatosis**  **(30%, n = 147)** | **No CAD**  **(51%, n = 244)** | **p-value^e^** |
| Patients, No. (%) | 90 (19) | 147 (30) | 244 (51) | < 0.001 |
| Years of age, mean (SD) | 67 (9.4) | 64 (10) | 76 (6.4) | < 0.001 |
| Women (vs. men), No. (%) | 57 (63) | 98 (67) | 172 (71) | > 0.05 |
| BMI, mean (SD)^a^ | 28 (4.8) | 28 (5) | 29 (5.4) | > 0.05 |
| Diabetes mellitus, No. (%)^b^ | 12 (13) | 27 (18) | 20 (8) | 0.011 |
| Hypertension, No. (%)^c^ | 55 (61) | 87 (59) | 81 (33) | p < 0.001 |
| Lipid-lowering drug, No. (%) | 46 (51) | 57 (39) | 39 (16) | p < 0.001 |
| Smoking, current, No. (%) | 15 (17) | 29 (20) | 20 (8) | 0.003 |
| Smoking, previous, No. (%)^d^ | 43 (47.8) | 54 (36.7) | 76 (31.2) | 0.019 |
| Creatinine clearance, mean (SD)^a^ | 85 (30.5) | 87 (31.8) | 105 (34.3) | < 0.001 |
| Cardiologist consultation, No. (%) | 28 (31) | 34 (23) | 62 (25) | > 0.05 |

* Completely inconclusive CCTA exams (n = 2) excluded from the comparison.

^a^ Excluding values of zero, BMI (kg/m^2^)(n = 3), creatinine clearance (mL/min per 1.73 m^2^)(n = 98).

^b^ Type I and II.

^c^ At least one blood pressure lowering drug.

^d^ Stopped smoking more than one month ago.

^e^ Tested with Pearsons’s Chi2, except for variables Years of age, BMI, Creatinine clearance which were tested by one-way ANOVA.
