# Supplementary material for: Coronary computed tomography angiography in primary care patients with chest pain or dyspnea – a cross-sectional study
Source: BMC Prim Care. 2025 May 20;26:178. doi: 10.1186/s12875-025-02877-z (PMC12090552; doi:10.1186/s12875-025-02877-z)
Supplement: Supplementary file 4 — Supplementary Material 4 [file 12875_2025_2877_MOESM4_ESM.docx]

### Supplementary Table 3. Subset analysis of patients ages 50-64 years (inclusion criterium in the SCAPIS: Swedish CArdioPulmonary bioImage Study [30]) with chest pain pre-test probability (PTP)

|  | **PTP % 5-15 (n = 92)** | **All (n = 148)** |
| --- | --- | --- |
| Suspected significant stenosis, No (%) | 11 (12) | 21 (14) |
| Atheromatosis, No (%) | 32 (35) | 53 (36) |
| No CAD, No (%) | 49 (53) | 73 (49) |
| PTP %, median (IQR) | 11 (6.5) | 11 (10) |
| Years of age, mean (SD) | 57 (4) | 57 (4) |
| Women (vs. men), No. (%) | 73 (79) | 96 (65) |
| BMI, mean (SD)^a^ | 28 (5) | 28 (5) |
| Diabetes mellitus, No. (%)^b^ | 8 (9) | 17 (12) |
| Hypertension, No. (%)^c^ | 39 (43) | 72 (49) |
| Lipid-lowering drug, No. (%) | 29 (32) | 45 (31) |
| Smoking, current, No. (%) | 11 (12) | 24 (16) |
| Smoking, previous, No. (%)^d^ | 32 (35) | 45 (31) |
| Creatinine clearance, mean (SD)^a^ | 103 (31) | 102 (30) |

^a^ Excluding values of zero, BMI (kg/m^2^) and creatinine clearance (mL/min per 1.73 m^2^).

^b^ Type I and II.

^c^ At least one blood pressure lowering drug.

^d^ Stopped smoking more than one month ago.
