# Supplementary material for: Coronary computed tomography angiography in primary care patients with chest pain or dyspnea – a cross-sectional study
Source: BMC Prim Care. 2025 May 20;26:178. doi: 10.1186/s12875-025-02877-z (PMC12090552; doi:10.1186/s12875-025-02877-z)
Supplement: Supplementary file 5 — Supplementary Material 5 [file 12875_2025_2877_MOESM5_ESM.docx]

### Supplementary Table 1. Missing data

| **Data coverage** | **CCTA**  **(n = 483)** | | **CCTA and eligible PTP**  **(n = 381)** | |
| --- | --- | --- | --- | --- |
|  | % with complete data (yes/no, ≠ 0 for continuous data) | % missing data | % with complete data (yes/no, ≠ 0 for continuous data) | % missing data |
| Years of age | 100 | 0 | 100 | 0 |
| Gender | 100 | 0 | 100 | 0 |
| BMI^a^ | 98.3 | 1.7 | 99.2 | 0.8 |
| Diabetes mellitus^b^ | 100 | 0 | 100 | 0 |
| Hypertension^c^ | 99.4 | 0.6 | 99.2 | 0.8 |
| Lipid-lowering drug | 97.5 | 2.5 | 97.6 | 2.4 |
| Smoking status^d^ | 99.8 | 0.2 | 99.7 | 0.3 |
| Creatinine clearance^a^ | 68.1 | 31.9 | 68.8 | 31.2 |
| Cardiologist consultation | 26.9 | 73 | 25 | 75 |

^a^ Excluding values of zero, BMI (kg/m^2^)(n = 3), creatinine clearance (mL/min per 1.73 m^2^)(n = 98).

^b^ Type I and II.

^c^ At least one blood pressure lowering drug.

^d^ Stopped smoking more than one month ago.
